# Targeting c-Met Receptor Overcomes TRAIL-Resistance in Brain Tumors

**DOI:** 10.1371/journal.pone.0095490

**Published:** 2014-04-18

**Authors:** Wanlu Du, Liubov Uslar, Sindhura Sevala, Khalid Shah

**Affiliations:** 1 Molecular Neurotherapy and Imaging Laboratory, Massachusetts General Hospital, Harvard Medical School, Boston, Massachusetts, United States of America; 2 Department of Radiology, Massachusetts General Hospital, Harvard Medical School, Boston, Massachusetts, United States of America; 3 Department of Neurology, Massachusetts General Hospital, Harvard Medical School, Boston, Massachusetts, United States of America; 4 Harvard Stem Cell Institute, Harvard University, Cambridge, Massachusetts, United States of America; Faculté de médecine de Nantes, France

## Abstract

Tumor necrosis factor related apoptosis-inducing ligand (TRAIL) induced apoptosis specifically in tumor cells. However, with approximately half of all known tumor lines being resistant to TRAIL, the identification of TRAIL sensitizers and their mechanism of action become critical to broadly use TRAIL as a therapeutic agent. In this study, we explored whether c-Met protein contributes to TRAIL sensitivity. We found a direct correlation between the c-Met expression level and TRAIL resistance. We show that the knock down c-Met protein, but not inhibition, sensitized brain tumor cells to TRAIL-mediated apoptosis by interrupting the interaction between c-Met and TRAIL cognate death receptor (DR) 5. This interruption greatly induces the formation of death-inducing signaling complex (DISC) and subsequent downstream apoptosis signaling. Using intracranially implanted brain tumor cells and stem cell (SC) lines engineered with different combinations of fluorescent and bioluminescent proteins, we show that SC expressing a potent and secretable TRAIL (S-TRAIL) have a significant anti-tumor effect in mice bearing c-Met knock down of TRAIL-resistant brain tumors. To our best knowledge, this is the first study that demonstrates c-Met contributes to TRAIL sensitivity of brain tumor cells and has implications for developing effective therapies for brain tumor patients.

## Introduction

c-Met, a transmembrane tyrosine kinase encoded by a c-met proto-oncogene [1], [2], has emerged as a key determinant of brain tumor growth [3]. c-Met is expressed in a wide variety of brain tumors, including glioblastoma multiforme (GBM) and medulloblastoma (MB), and its expression level frequently correlates with tumors histological grade and poor patient prognosis [4]. Overexpression or abnormal activation of c-Met in tumor cells leads to induction of proliferation, migration, and invasion along with the inhibition of apoptosis. Conversely, downregulation of c-Met in experimental human tumor xenografts leads to inhibition of tumor growth [5]. Different approaches to inhibit c-Met and its downstream signaling pathways have recently been developed, including small molecular tyrosine kinase inhibitors [6], which are currently undergoing clinical trials in cancer patients.

Tumor necrosis factor related apoptosis-inducing ligand (TRAIL) is a pro-apoptotic protein that specifically targets tumor cells both *in vitro* and *in vivo* [7]–[10], thus making it a very promising candidate for future cancer therapy. TRAIL induces apoptosis by binding to its cognate death domain-containing receptors (DR) 4 and 5 on the cell surface [11], [12], which then leads to a cascade of death-inducing signaling complex (DISC) formation, caspase activation and ultimately the execution of the apoptotic program [13]–[15]. We and others have previously shown that stem cell-based delivery of S-TRAIL is highly efficacious in orthotopic brain tumor models due to the tumoritropic properties of SC that lead to on-site, sustained release of therapeutic S-TRAIL [16]–[19]. However, tumor cells have varying response to TRAIL mediated killing and understanding the molecular mechanisms involved in TRAIL resistance in tumor cells becomes a pre-requisite for the broader successful application of TRAIL based therapies in the future. Recently, abnormalities in c-Met signaling have been reported to correlate with drug resistance in patients with cancer [20]. However, the molecular mechanism for the role of c-Met in TRAIL resistance in brain tumors remains unknown.

In the present study, we first screened a panel of brain tumor lines for their sensitivity to S-TRAIL. In an effort to develop anti-brain tumor therapies that overcome TRAIL resistance, we explored the interaction between c-Met and DRs and their contribution in TRAIL resistance in brain tumors. Furthermore, we addressed the effect of targeting c-Met by lentivirial-mediated expression of shRNA in combination with mesenchymal stem cell (MSC)-delivered a secretable form of TRAIL (S-TRAIL) in MSC-tumor cell co-cultures *in vitro* and *in vivo*.

## Materials and Methods

### Cell culture and viral transduction

Medulloblastoma (MB) cell lines (UW426, R262, R300, DAOY, UW473) from American Type Culture Collection (ATCC) [21] were cultured in Dulbecco's modified Eagle's medium (DMEM) (Invitrogen/GIBCO, Grand Island, NY) supplemented with 10% fetal calf serum (Valley Biomedical Inc. Winchester, VA), 1% Penicillin/Streptomycin (Invitrogen, Carlsbad, CA), 2% sodium bicarbonate (GIBCO), 1% nonessential amino acids (Mediatech Inc, Manassas, VA), and 1% sodium pyruvate (Cellgro, Manassas, VA). Established human glioma lines (LN229, LN319, U138, U373, U87, U251 and Gli36) and primary human glioma lines (GBM4 and GBM8) were grown as previously described [18], [22]. Murine mesenchymal stem cells (MSC) (kindly provided by Dr. Darwin Prockop, University of Texas) were grown as previously described [18]. Lentiviral (LV)-Fluc-mCherry (Fmc), LV-scrambled (scr), LV-shMet (kindly provided by Jeffrey Engelman, Massachusetts General Hospital Cancer Center, Boston, MA) constructs were packaged in 293T cells as described previously [23]. UW473 cells were infected with LV-scr or LV-shMet, and then selected with puromycin, cultured for 1∼2 passages, and infected again with LV-Fmc. The double infected cells were then subjected to fluorescence activated cell sorting (FACS Aria Cell-Sorting System, BD Biosciences) and UW473scr-Fmc and UW473shMet-Fmc lines were created. MSC-GFP and MSC-S-TRAIL cell lines were created through retroviral transduction as described previously [24].

### In vitro cell viability assay

Human 293T cells were transduced with lentiviral vector LV-S-TRAIL [25] at an MOI of 1 and 36 hours later, a culture medium and the cells were harvested. Proteins were isolated from harvested cells, resolved by sodium sodium dodecyl sulfate-polycrylamide gel electrophoresis (SDS-PAGE), and immunoblotted with anti-TRAIL antibody (ProScience, Poway, CA). S-TRAL concentration in the conditioned culture medium was measured by ELISA with the TRAIL Immunoassay Kit (Biosource International, Camarillo, CA) according to the manufacturer's protocol, using recombinant human TRAIL expressed in Escherichia coli as a standard. Tumor cells (0.5×10^4^/well) were seeded in 96-well plates and incubated with varying concentrations (0∼1000 ng/ml) of S-TRAIL obtained from transduced 293T cells for 24 h. Cell viability was measured by a quantitative luminescence assays using an ATP-dependent luminescent reagent (CellTiter-Glo, Promega, Madison, WI) according to the manufacturer's instructions. Based on our previously published screening of glioma cells to TRAIL mediated apoptosis, we chose TRAIL sensitive and resistant glioma lines [26]. TRAIL sensitivity and resistance was determined by treating different glioma cells with different concentrations of S-TRAIL and glioma cell viability was determined 24 h post-TRAIL treatment. For c-Met inhibition studies, tumor cells were incubated with c-Met inhibitor PHA665752 (1 uM) (Selleck Chemicals, Houston, TX) for 12 h before TRAIL treatment. All experiments were performed in triplicates.

### Western blotting and immunoprecipitation

Following treatment, MB cells were lysed with NP40 buffer supplemented with a protease inhibitor mixture (Complete Mini EDTA-free, Roche Applied Science, Indianapolis, IN) and phosphatase inhibitors II/III (Sigma, St Louis, MO). 30 µg of harvested proteins from each lysate were resolved on 10% SDS-PAGE, and immunoblotted with antibodies against DR5, DR4 (ProSci, Inc., Poway, CA), c-Met, caspase 8, cleaved caspase 3, cleaved poly (ADP-ribose) polymerase (PARP), FLICE inhibitory protein (FLIP) (Cell Signaling, Beverly, MA), or α-tubulin (Sigma); and detected by chemiluminescence after incubation with HRP-conjugated secondary antibodies (Santa Cruz). Quantification of western blot signals was performed using Image J. The data was normalized to α-tubulin expression. For immunoprecipitation, whole cell lysates were pre-incubated with Protein G Resin (GenScript, Piscataway, NJ) for 3 h at 4°C to exclude nonspecific binding to Protein G-Sepharose. After centrifugation, supernatants containing proteins were added with IgG (Santa Cruz) or indicated antibodies, rotated over night at 4°C, and then added with Protein G-Sepharose for 3 h. The immune complex was spun down, washed, resuspended in SDS-gel sample buffer and boiled at 100°C for 5 min. Immunoprecipitates were separated by SDS-PAGE and immunoblotted with indicated antibodies.

### MB cells and MSC co-culture experiments and cell viability assays

For co-culture experiments, UW473scr-Fmc or UW473shMet-Fmc cells were plated with varying numbers of MSC-GFP or MSC-S-TRAIL (10∶1 or 5∶1) in a 96-well plate, incubated for 36 hours and then cell viability was measured by a quantitative luminescence assays using an ATP-dependent luminescent reagent (CellTiter-Glo, Promega), according to the manufacturer's instructions.

### Intracranial cell implantation and in vivo imaging

UW473scr-Fmc, UW473shMet-Fmc, MSC-GFP, and MSC-S-TRAIL cells were harvested at 80%–90% confluence and implanted in various combinations as the following experimental groups: UW473scr-Fmc with MSC-GFP, UW473scr-Fmc with MSC-S-TRAIL, UW473shMet-Fmc with MSC-GFP, UW473shMet-Fmc with MSC-S-TRAIL. These combinations were implanted stereotactically into nude mice brains (3×10^5^ tumor cells/mouse and 6×10^4^ MSCs/mouse) in the following co-ordinates: 2.2 mm lateral from bregma, 2.5 mm ventral from dura on the cranial suture. Mice were imaged for Fluc activity on 1, 3 and 7 days after implantations as previously described [18]. All *in vivo* procedures were approved by the Subcommittee on Research Animal Care at Massachusetts General Hospital.

### Tissue processing and immunohistochemistry

Mice were perfused by injecting ice-cold 4% paraformaldehyde (PFA) directly into the heart and the brains were fixed in 4% PFA and frozen sections were obtained for immunohistochemistry. For cleaved caspase 3 staining, sections were incubated for 1 hour in a blocking solution (0.3% BSA, 8% goat serum and 0.3% Triton-X100) at room temperature, followed by incubation at 4°C overnight with anti-cleaved caspase 3 antibody (Cell Signaling) diluted in blocking solution. Sections were incubated in Alexa Fluor 647 goat anti-rabbit secondary antibody (Invitrogen), and visualized using confocal microscope (LSM Pascal, Zeiss). The number of cleaved caspase 3 positive cells was calculated by counting positive cells in randomly selected field of views under a microscope (20×).

### Statistical analysis

Data were analyzed by Student's *t*-test when comparing two groups and by ANOVA when comparing more than two groups. Data are expressed as mean ± s.e.m., and differences were considered significant at *P*<0.05.

## Results

### Knock down c-Met protein sensitizes resistant brain tumor cells to TRAIL-induced apoptosis

**Figure 1:**
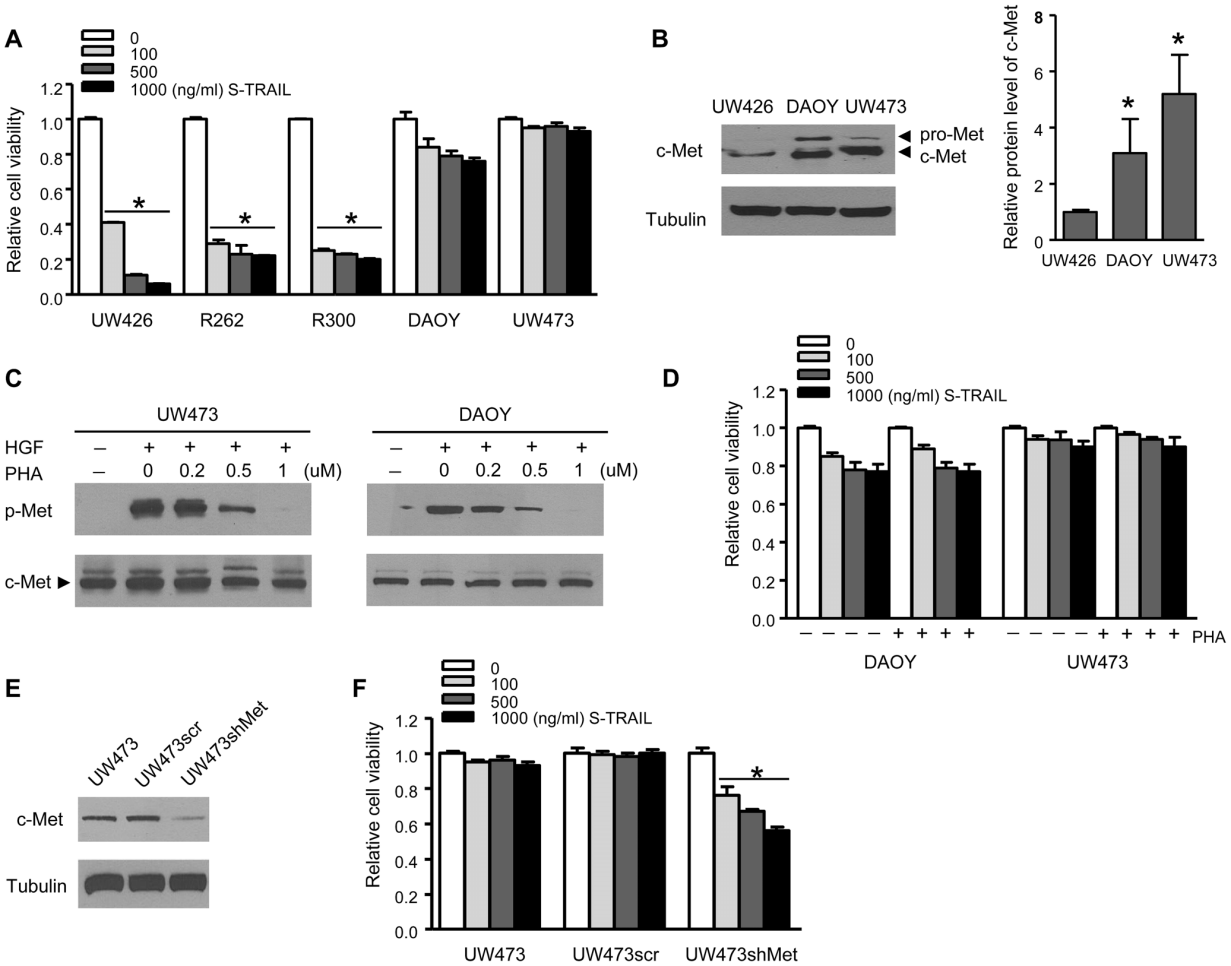
Knock down of c-Met protein sensitizes resistant medulloblastoma (MB) cells to S-TRAIL treatment. (A) MB cell viability analysis showing the effect of varying concentrations of S-TRAIL treatment (24 h) on different MB cell lines. (B) Left, Western blot analysis of c-Met and tubulin levels in indicated MB cell lines. Right, plot showing the statistic results of c-Met protein levels in the MB lines. (C) MB lines UW473 and DAOY were pre-incubated with c-Met inhibitor (PHA665752) before treatment with HGF (25 ng/ml). Protein lysates were then immunoblotted for phosphorylated (p-Met) or total c-Met. (D) Cell viability analysis showing the effect of S-TRAIL treatment on both DAOY and UW473 cells with (+) or without (−) the presence of PHA665752 (PHA). (E) Western blot analysis c-Met and tubulin levels in UW473 cells stable-infected with LV-scrambled (UW473scr) or LV-shMet virus (UW473shMet). (F) Cell viability analysis showing the effect of S-TRAIL treatment (24 h) on UW473, UW473scr, or UW473shMet respectively. * denotes *P*<0.05.

**Figure 2:**
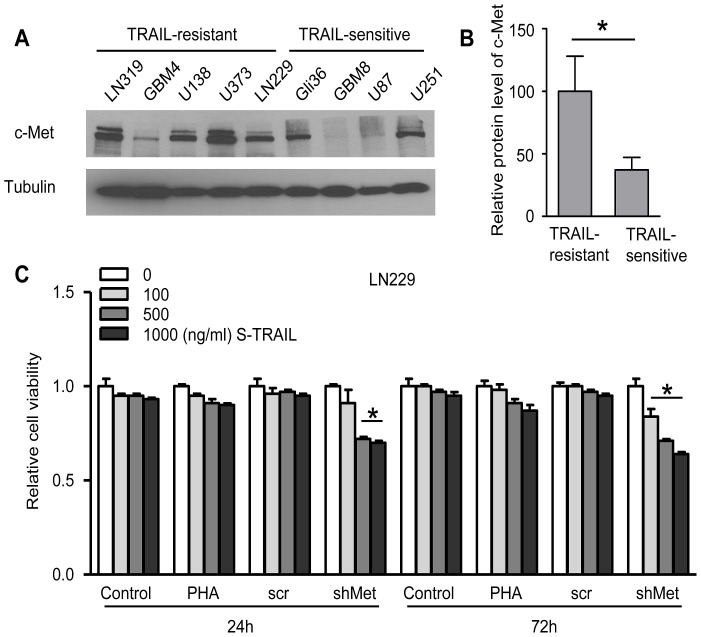
Knock down of c-Met protein sensitizes resistant glioma cells to S-TRAIL treatment. (A) Western blot analysis of c-Met and tubulin levels in indicated glioma lines. (B) Plot showing the statistic results of c-Met protein levels in both TRAIL-resistant and TRAIL-sensitive glioma lines. (C) LN229 cell line viability analysis showing the effect of S-TRAIL treatment (24 h and 72 h) on indicated groups. * *P*<0.05.

In order to investigate the relationship between c-Met protein and TRAIL resistance in brain tumor cells, we first tested a panel of human medulloblastoma (MB) cell lines for their sensitivity to S-TRAIL treatment. While UW473 and DAOY lines demonstrated resistance to various concentrations of S-TRAIL ranging from 100∼1000 ng/ml, UW426 line exhibited most sensitivity to these concentrations (Fig. 1A). PI/Annexin V dual staining combined with flow cytometry analysis revealed that UW426 line exhibited 3-fold increase in apoptotic (Annexin V positive) cell population when treated with S-TRAIL for 12 h, whereas no significant increase in the apoptotic population was found in DAOY or UW473 lines (Figure S1). In both of the TRAIL-resistant cell lines, UW473 and DAOY, c-Met protein expression levels were significantly higher than that of TRAIL-sensitive line UW426 (Fig. 1B). Therefore to further study the role of c-Met in TRAIL-resistance of MB cells, we used two strategies to manipulate c-Met protein and its related signaling pathways. First, PHA665752, a potent and highly selective c-Met inhibitor, was used to block c-Met activation in MB cells. Western blot analysis showed PHA665752 inhibited HGF-induced c-Met phosphorylation in a dose dependent manner (Fig. 1C). Both TRAIL-resistant UW473 and DAOY lines treated with different concentrations of S-TRAIL with or without the presence of PHA665752 showed no change in cell viability (Fig. 1D). Second, UW473 cell line was stably transfected with lentiviral (LV) constructs expressing c-Met-specific shRNA (LV-shMet) or scrambled control (LV-scr). As revealed by the immunoblot analysis, the c-Met protein level was significantly decreased in UW473 expressing shMet compared to the scrambled control (Fig. 1E). Although knocking down c-Met protein has no effect on the cell viability (Figure S2A), S-TRAIL treatment resulted in a significant decrease in cell viability in UW473shMet cells as compared to controls (Fig. 1F). We further examined c-Met protein levels among both TRAIL resistant and TRAIL sensitive GBM lines obtained from our previous screening [26]. We found that c-Met expression level was significantly higher in TRAIL resistant lines as compared with that of TRAIL sensitive lines (Fig. 2A–B). In a highly TRAIL resistant line, LN229, pre-incubation with the c-Met inhibitor, PHA665752 again showed no effect on TRAIL sensitivity, while knocking down c-Met protein levels via stable expression of c-Met specific shRNA (shMet) greatly potentiated TRAIL sensitivity (Fig. 2C). Taken together, the above results showed that knock-down of c-Met protein level, but not inhibition of c-Met activation, sensitized TRAIL-resistant brain tumor cells to TRAIL treatment.

### c-Met interacts with DR5 and affects DISC formation upon TRAIL stimulation

**Figure 3:**
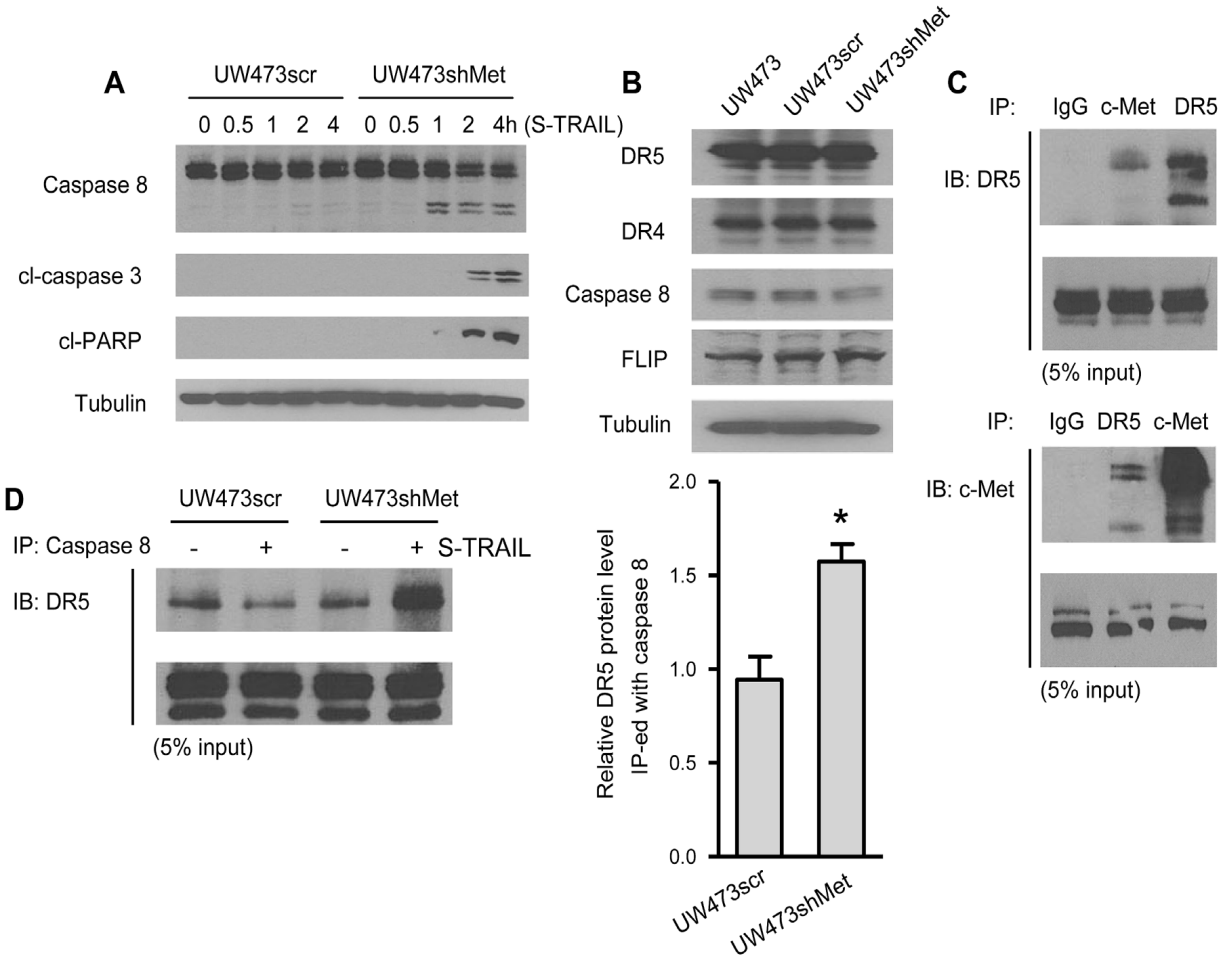
c-Met interacts with DR5 and affects DISC formation upon S-TRAIL stimulation. (A) Western blot analysis of caspase 8, cleaved (cl)-caspase 3, cleaved (cl)-PARP, and tubulin levels in UW473scr and UW473shMet cells upon S-TRAIL stimulation for indicated time periods. (B) Western blot analysis showing DR5, DR4, caspase 8, FLIP, and tubulin levels in UW473, UW473scr and UW473shMet cells. (C) Upper panel, Western blot analysis showing the DR5 levels in the immunoprecipitates of IgG, anti-c-Met, and anti-DR5 (5% of each input was also blotted for total DR5 as control); Lower panel, Western blot analysis showing the c-Met levels in the immunoprecipitates of IgG, anti-DR5 and anti-c-Met (5% of each input was also blotted for total c-Met as control). (D) Left panel, Western blot analysis showing DR5 levels in the immunoprecipitates of anti-caspase 8 from UW473scr or UW473shMet cells with (+) or without (−) the presence of S-TRAIL stimulation (5% of each input was also blotted for total DR5 as control). Right panel, plot showing the quantitative analysis of the immunoblots on the left. * denotes *P*<0.05.

We further explored the mechanism underlying the role of c-Met protein in TRAIL-resistance of brain tumor cells. As the UW473 line is fully resistant to TRAIL, we utilized this line for further studies. Western blot analysis showed that UW473shMet line had a stable knock-down of c-Met levels compared to UW473scr or UW473 lines (Figure S3). Next, we examined the protein levels of caspase 8, cleaved caspase 3 and cleaved poly (ADP-ribose) polymerase (PARP) in both UW473scr and UW473shMet cells upon S-TRAIL stimulation for varying amounts of time (Fig. 3A). A time dependent activation of caspase 8, followed by the cleavage of both caspase 3 and PARP were seen in UW473shMet cells but not in UW473scr cells. Since c-Met has the potential to complex with and modulate DRs independent of its classical tyrosine kinase driven second messenger responses [27], further immunoblot analysis showed that there was no significant change in the levels of DR5, DR4, caspase 8 and FLICE-like inhibitory protein (FLIP) in UW473shMet, UW473 and UW473scr cells (Fig. 3B). However, a reciprocal immunoprecipitation assay demonstrated an interaction of c-Met with DR5 (Fig. 3C). This finding led us to explore whether downregulation of c-Met alters DISC formation. Both UW473scr and UW473shMet cells extracts with or without S-TRAIL treatment for 2 h were subjected to immunoprecipitation with anti-caspase 8 antibody, precipitated complexes and then analyzed by western blot using antibodies against DR5 (the main DR isoform found on MB cell membrane according to our FACS analysis). The protein level of DR5 among the precipitated DISC components was greatly increased in S-TRAIL treated UW473shMet cells compared to UW473scr cells (Fig. 3D), indicating that the DISC formation was more efficient in UW473shMet cells upon S-TRAIL stimulation. These results suggest that c-Met interacts with DR5 and affects DISC formation upon TRAIL treatment.

### Knock down of c-Met sensitizes TRAIL resistant brain tumor cells to stem cell-delivered S-TRAIL

**Figure 4:**
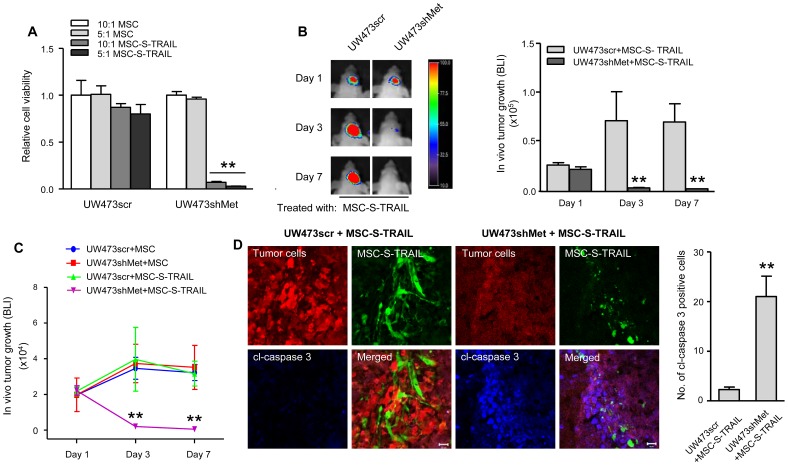
Knock down of c-Met sensitizes resistant MB cells to stem cell-delivered S-TRAIL both *in vitro* and *in vivo*. (A) Cell viability measured by Fluc signal showing the effects of MSC-derived S-TRAIL in the co-cultures of MSC or MSC-S-TRAIL with UW473scr-Fmc (UW473scr), or UW473shMet-Fmc (UW473shMet) cells at various culturing ratios. (B) Left panel: Fluc bioluminescence imaging (BLI) on days 1, 3 and 7 of mice intraparenchymally implanted with UW473scr or UW473shMet cells admixed with MSC-S-TRAIL. Representative images of mice for each group are shown. Right panel, statistical analysis of Fluc bioluminescence activity observed in respective groups of mice represented on the left. (C) Plot showing Fluc bioluminescence intensities (representative of *in vivo* tumor growth). (D) Left, the brain sections from the indicated groups (UW473scr+MSC-S-TRAIL or UW473shMet+MSC-S-TRAIL) stained with cl-caspase 3 (blue) and imaged together with mCherry (red, represents tumor cells) and GFP (green, represents MSC secreting S-TRAIL). Right panel, plot representing the number of calculated activated caspase 3 positive cells in the corresponding samples on the left panel (scale bar  =  20 µm). ** denotes *P*<0.01.

To investigate whether c-Met knock down can sensitize brain tumor cells to TRAIL treatment *in vivo*, we first engineered *in vivo* imageable UW473scr and UW473shMet cells by transducing them with LV-Fluc-mCherry (Fmc). A direct correlation between Fluc signal and cell number was seen within the ranges tested in both cell lines (Figure S2B). Additionally, we used engineered MSC lines expressing either S-TRAIL (MSC-S-TRAIL) or GFP (MSC) [24]. MSC or MSC-S-TRAIL co-cultured with either UW473scr-Fmc or UW473shMet-Fmc in different ratios and the viability of UW473 cells was assessed by Fluc bioluminescence imaging. A significant decrease in cell viability was seen when UW437shMet-Fmc cells co-cultured with MSC-S-TRAIL compared with the control (UW473scr-Fmc co-cultured with MSC-S-TRAIL) (Fig. 4A). Next, we implanted UW473scr-Fmc or UW473shMet-Fmc cells and MSC expressing only GFP or S-TRAIL and GFP intracranially in nude mice and then imaged the tumor cell growth over time. Serial Fluc bioluminescence imaging revealed a significant inhibition of tumor cell growth in mice when treated with MSC-S-TRAIL in UW437shMet-Fmc cells (Fig. 4B and C). Furthermore, a significantly increased number of cleaved caspase 3 positive cell was observed in brain sections obtained from the mice implanted with UW473shMet-Fmc and MSC-S-TRAIL compared with that of UW473scr-Fmc and MSC-S-TRAIL (Fig.4D), showing the involvement of caspase-mediated apoptosis and further confirming that knock down c-Met overcomes TRAIL-resistance of brain tumor cells *in vivo*. These *in vivo* results further confirmed that knock down of c-Met does overcome TRAIL-resistance of brain tumor cells and provided evidence for the involvement of caspase-mediated apoptosis in this process. Taken altogether, the *in vitro* and *in vivo* results in this study support each other in the notion that knock down of c-Met sensitizes TRAIL resistant brain tumor cells to MSC-S-TRAIL treatment.

## Discussion

In this study, we investigated the involvement of c-Met protein in TRAIL resistance and further identified the interaction between c-Met and DR5 in brain tumor cells. We show that down-regulation of c-Met potentiates DISC formation, and sensitizes TRAIL resistant brain tumor cells to TRAIL mediated apoptosis both *in vitro* and *in vivo*.

Much of the research till date involving TRAIL signaling has focused on its usage in cancer treatment; however, an increasing number of publications are starting to report on the predominance of TRAIL resistance in primary human tumor cells [10], which require sensitization for the induction of TRAIL-based apoptosis. Although, sensitization of cancer cells by treatment with chemotherapeutic drugs and irradiation has been shown to restore TRAIL sensitivity in many TRAIL-resistant brain tumor cells [28], none of the previous studies have explored the role of c-Met down-regulation in sensitizing TRAIL resistant tumor cells to TRAIL mediated apoptosis. Previous studies have also show that c-Met inhibitor synergizes with TRAIL to induce papillary thyroid carcinoma (PTC) cell death, most likely through inhibition of abnormal c-Met activation and upregulation of DR5 [29]. As mentioned earlier, c-Met inhibitors function by inhibiting abnormal c-Met activation, as opposed to affecting c-Met protein levels, which can be observed through a change in p-Met levels. In contrast to the high expression levels of p-Met in PTC cells, the basal p-Met protein levels were extremely low in the brain tumor lines we tested, which suggests that c-Met protein itself, independent of its activation, might play a role in TRAIL resistance of brain tumor cells. We thus exerted our efforts to elucidate the involvement of c-Met protein in the TRAIL-related apoptosis pathway. In contrast to the previous studies which suggest that the dysregulation of HGF/c-Met pathway may affect TRAIL-sensitivity via upregulation of DR5 level [30], we found that both the levels of DR4 and DR5 protein were unaltered after knocking down c-Met in TRAIL-resistant brain tumor cells. In addition, co-immunoprecipitation experiments reveal that c-Met interacts with DR5 and affects DISC formation most likely through the same mechanism implicated in the interaction of c-Met with Fas receptor, which sequestered Fas receptor and prevented its self-aggregation and its downstream apoptosis execution, as previously reported [31], [32]. Based on our results, we hypothesize that the interaction between c-Met and DR5 sequesters DR5 and prevents its self-aggregation and its downstream apoptosis execution upon TRAIL stimulation. Knock down of c-Met protein greatly augments TRAIL-induced cell apoptosis by freeing DR5 from the complex. Hence, our results suggest a new mechanism for c-Met mediated TRAIL resistance and provide immense evidence for the utility of a mechanism based combination therapy for targeting brain tumors.

The limited availability of non-invasive methods to monitor multiple molecular events has been one of the main limitations in testing the efficacy of various tumor therapy paradigms. In our previous studies, we have shown that we can follow delivery of NSC [23], MSC [18] and quantify tumor burden *in vivo* [23], [25] using non-invasive bioluminescence imaging. In the current studies, we have labeled tumor cells and stem cells with bimodal imaging markers (bioluminescent and fluorescent) expressed as a single transcript, which allows for expanding the number of events that can be visualized in real-time *in vivo* and efficiently applied bioluminescence imaging to follow tumor cell fate *in vivo*. Although the optical imaging methods utilized in this study are ideally suited for pre-clinical studies, further studies validating this approach incorporating clinical imaging modalities like MRI will ease the translation of our approach into clinics.

In conclusion, our studies propose for the first time that c-Met plays a role in TRAIL-resistance of brain tumors by interacting with DR5 (without changing DR5 levels) and affecting DISC formation and subsequently TRAIL-mediated apoptosis execution both *in vitro* and *in vivo*. Our findings provide preclinical evidence that c-Met itself, independent of its activation, is involved in this mechanism of TRAIL-resistance. Even though further testing needs to be performed to elucidate the intricacies of this mechanism possibly through the utilization of additional c-met inhibitors and other methods to target c-Met expression, we believe that the current study has taken the first steps toward shedding the light on a new mechanism of TRAIL resistance in brain tumors.

## Supporting Information

Figure S1 **Induction of apoptosis by S-TRAIL treatment in MB cell lines.** Effects of S-TRAIL treatment on UW426, DAOY and UW473 lines. Numbers in the respective quadrants indicate the percentage of cells presents in this area. In UW426 cells, the Annexin V positive cell population was significantly increased upon S-TRAIL treatment. * *P*<0.05, ** *P*<0.01.
(TIF)

Figure S2 **Characterization of modified UW473 lines.** (A) Cell viability analysis showing the growth rate of both UW473scr-Fmc and UW473shMet-Fmc cells. (B) Top, representative fluorescent images of both UW473scr-Fmc and UW473shMet-Fmc cells. Bottom, plots showing the Fluc intensities of modified tumor cells with different cell numbers.
(TIF)

Figure S3 **The c-Met protein levels in UW473 lines stably transduced with LV-scrambled or LV-shMet.** Western blot analysis of c-Met and tubulin levels in UW473 cells stably transduced with LV-scrambled (UW473scr) or LV-shMet (UW473shMet).
(TIF)
