# Supplementary figures and images for: Targeting c-Met Receptor Overcomes TRAIL-Resistance in Brain Tumors
Source: PLoS One. 2014 Apr 18;9(4):e95490. doi: 10.1371/journal.pone.0095490 (PMC3991662; doi:10.1371/journal.pone.0095490)

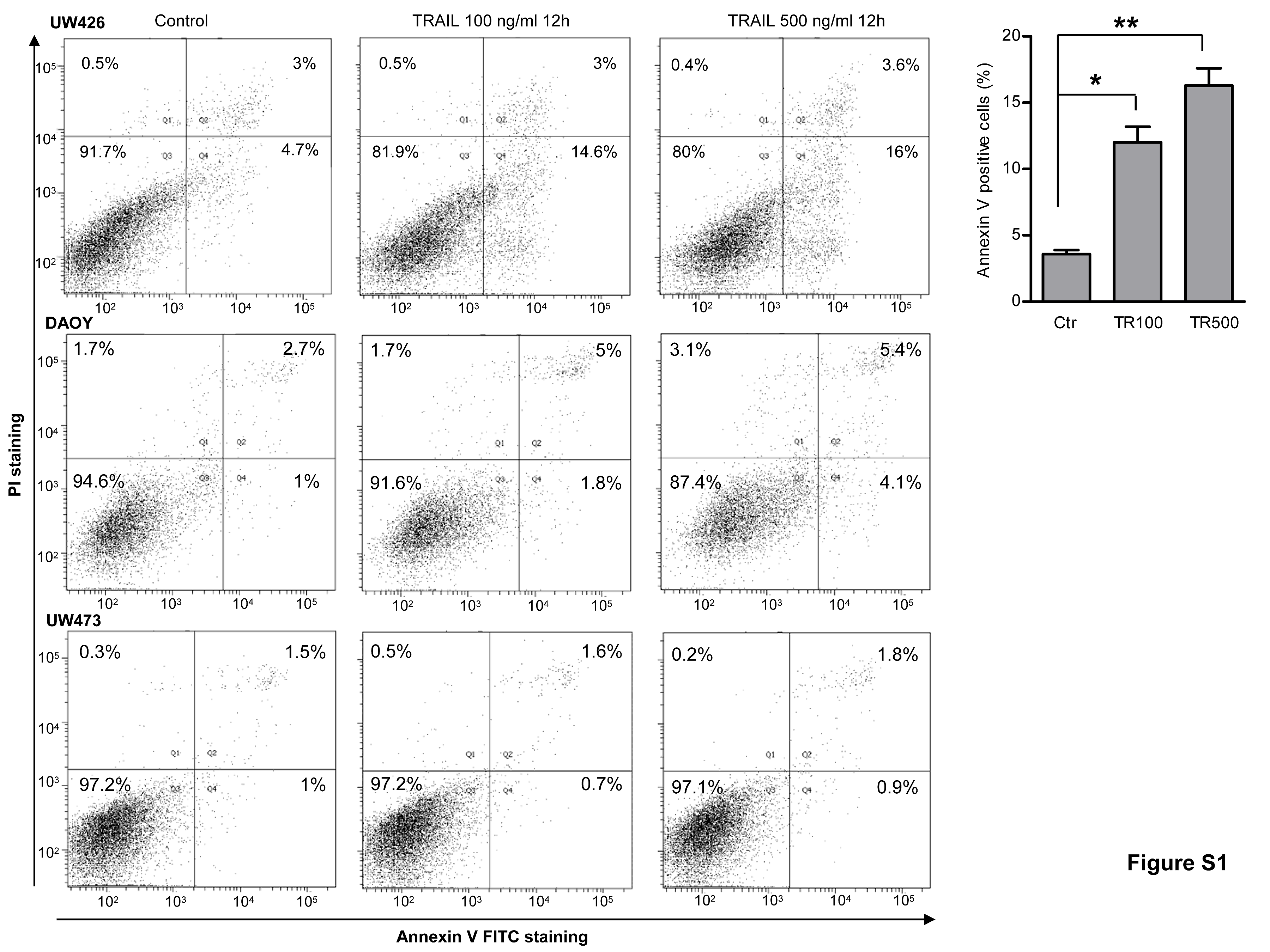

Supplement: Figure S1 — Induction of apoptosis by S-TRAIL treatment in MB cell lines. Effects of S-TRAIL treatment on UW426, DAOY and UW473 lines. Numbers in the respective quadrants indicate the percentage of cells presents in this area. In UW426 cells, the Annexin V positive cell population was significantly increased upon S-TRAIL treatment. * P<0.05, ** P<0.01. (TIF) [file pone.0095490.s001.tif]

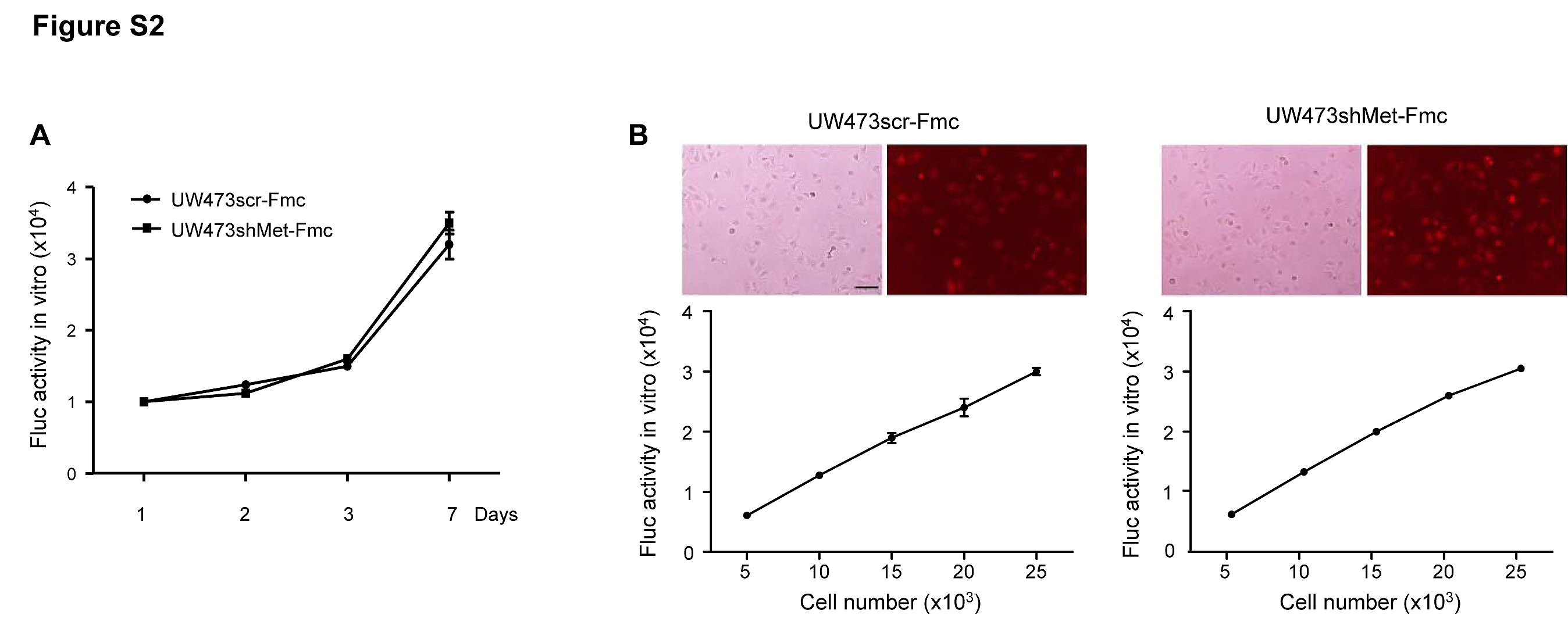

Supplement: Figure S2 — Characterization of modified UW473 lines. (A) Cell viability analysis showing the growth rate of both UW473scr-Fmc and UW473shMet-Fmc cells. (B) Top, representative fluorescent images of both UW473scr-Fmc and UW473shMet-Fmc cells. Bottom, plots showing the Fluc intensities of modified tumor cells with different cell numbers. (TIF) [file pone.0095490.s002.tif]

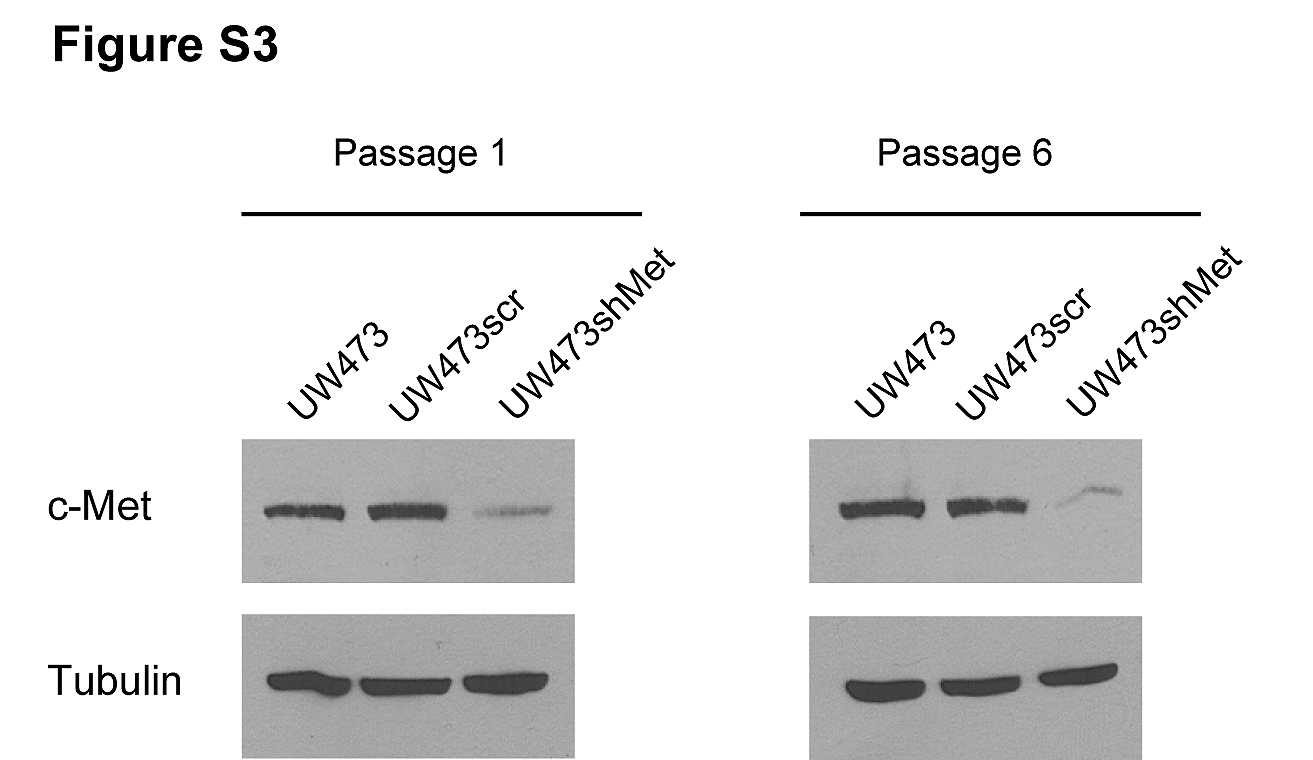

Supplement: Figure S3 — The c-Met protein levels in UW473 lines stably transduced with LV-scrambled or LV-shMet. Western blot analysis of c-Met and tubulin levels in UW473 cells stably transduced with LV-scrambled (UW473scr) or LV-shMet (UW473shMet). (TIF) [file pone.0095490.s003.tif]
